# The Significance of Experiencing Clinical Responsibilities for Specialty Career Choice

**DOI:** 10.1007/s40670-019-00832-z

**Published:** 2019-10-28

**Authors:** Sophie Querido, Marlies De Rond, Lode Wigersma, Sjoukje van den Broek, Olle ten Cate

**Affiliations:** 1 Central Board for Specialty training in Elderly Care Medicine in the Netherlands (SOON), P.O. Box 19025, 3501 DA Utrecht, The Netherlands; 2 Center for Research and Development of Education, University Medical Center Utrecht, Utrecht, The Netherlands; 3 Royal Dutch Medical Association (KNMG), Utrecht, The Netherlands; 4 Dutch Association of Public Health Physicians (VAV), Utrecht, The Netherlands; 5 Center of Education and Training, Unit of Medical Education, University Medical Center Utrecht, Utrecht, The Netherlands

**Keywords:** Career preference, Clinical responsibility, Specialty choice, Qualitative interview study

## Abstract

**Aim:**

Medical trainees make career choices in the final year of medical school or after graduation, if they do not continue with residency directly. Most Dutch medical students are trained in vertically integrated (VI) curricula, with early clinical experience and a gradual increase in clinical responsibilities. Students in such curricula have been reported to make career choices at an earlier stage than graduates from more traditionally designed curricula. Many Dutch graduates build further clinical experience after graduation as physicians-not-in-training (PNITs) before beginning residency. We explored how students make career choices and whether pre-residency clinical responsibilities influence this choice.

**Method:**

A qualitative study with a phenomenology approach was used. The authors conducted a longitudinal interview study of medical students with two intervals over a 2-year period. The interview questions covered how trainees establish career preferences and which factors affect preference and choice over time.

**Results:**

Experiencing clinical responsibility was a key factor for career preference during all interview rounds. Being a PNIT who makes diagnostic and therapeutic decisions, have their own patients and have significant patient care responsibilities creates opportunities to build an image of a future context of employment. Some participants mentioned that their experience of having full responsibility as a PNIT was pivotal in a career preference change.

**Conclusion:**

Clinical responsibility as a student or a PNIT appears to be important for career preference and choice. The experience of responsibility as a medical doctor forces trainees to reflect on personal needs and to consider which career preference fits best.

## Introduction

Medical trainees need to make an important career choice about a specialty when they face the transition from medical school to residency. When they feel urged to make this choice depends on the structure and length of medical training in the country [1].

In the Netherlands, the duration of undergraduate medical education is usually 6 years and includes 2 years to 4 years of clerkships. Most students are admitted directly from secondary school [2]. After graduation, the medical graduate can either do temporary supervised clinical work before residency as a so-called physician-not-in-training, start a PhD trajectory or start residency directly [1–5]. Many of the Dutch medical graduates choose physician-not-in-training work experience in one or more specialties before residency to make a thoughtful specialty decision [5]. In the United Kingdom (UK), all junior doctors have a 2-year foundation training after medical school and choose a specialty in the second year, which is at least 18 months after finishing medical school [1]. Many UK junior doctors consider this period too short to make a well-founded career choice, because of insufficient clinical experience [6].

Worldwide, graduates have preferences for popular specialties, such as surgery, gynaecology and dermatology, while other specialties show a shortage of interest including psychiatry and primary care [7–10]. Dutch graduates apply for a residency in an open job market system, rather than a national matching model; many preferences focus on a limited number of popular specialties, with a consequent shortage of available positions. Dutch graduates usually take an interval period of months up to a year (or even longer) to gain clinical or research experience, often to optimize chances of being selected, especially for popular specialties [2–5]. This, in turn, allows programs to raise acceptance criteria, in a self-reinforcing process that further increases the interval between undergraduate and postgraduate training as graduates need time to meet these criteria. Throughout the year, approximately 2400 Dutch medical students graduate and become eligible to start residency [11, 12]. Graduates who do not start postgraduate medical training directly can roughly be categorized in three groups: (1) those serving in patient care to gain clinical experience as a physician; we will categorize them as ‘physicians-not-in-training’ (PNITs). This group is approximately 3736 physicians [5]; (2) those starting or completing a PhD trajectory, often in a direction aligning with their specialty preference. This group is approximately 1366 physicians [5]; and (3) those employed in a non-clinical area. This group is approximately 412 physicians [5]. There is no nationally coordinated placement, and most of the physicians start residency after a period of experience as a PNIT or PhD student [5]. This is quite a usual pathway for Dutch graduates.

In the period between 1975 and 2000, all Dutch medical schools have gradually developed integrated curricula, both horizontally, i.e. among the basic sciences, and vertically, intertwining clinical topics and experiences with basic sciences. Vertical integration includes a gradual increase in clinical responsibilities [13]. An important feature of vertical integration is that clinical experience, such as clerkships, is offered early in the program [2, 13, 14].

Another feature is that students are given increased responsibilities in patient care, especially in the final year of medical school [15–18]. Students in most final program years of Dutch medical curricula (the ‘transitional year’) are called ‘semi-physicians’—not students—and must do clinical ward work at the level of a junior resident, with a limited number of patients and adequate supervision [2, 19–21]. A survey study among graduates of two cohorts, one from traditional and the other from vertically integrated curricula, showed that graduates from the latter had made the definite career choice at an earlier stage and needed less time and fewer applications to obtain a residency position than those from the traditional, less vertically integrated, curricula [21]. This study did not, however, provide insight into the factors determining this positive effect of early career choice in vertically integrated curricula.

In our study, we aimed to explore how students from vertically integrated curricula develop career preferences and make their career choice and whether additional pre-residency clinical experiences and increased responsibilities influence this choice.

## Method

### Methodology

To appreciate the development of career preferences and career choice over time, we designed a longitudinal interview study. Longitudinal research can provide a deeper understanding of how medical students develop a career preference over time and which factors influence career preference. There is a paucity of longitudinal studies focusing on the preference for medical specialties in general. The only longitudinal studies to date are specialty specific and are focused on primary care [10] and surgery [22]. Other longitudinal research focuses on student interest but lacks explanation of the changes of interest and uses written surveys [23–25]. One recent longitudinal study performed in the UK found that when considering groups of specialties, two thirds of graduates had a stable specialty preference since year 4 of medical school. Specialty choice was reported to be strongly influenced by experiences within the specialty at medical school and during the two foundation years [6]. However, the UK medical education structure differs from that of other countries, such as the Netherlands [1], and therefore, more research is required.

We conducted this longitudinal, interview-based qualitative study to gain insight into the influence of clinical responsibility on the career preference and career choice at three different moments [26]. We used a phenomenological approach, as this allows for interpretation of data, extraction of meaning and understanding of data to develop deeper understanding of the concept. Phenomenology attempts to understand how individuals construct meaning from their experiences through the perception of events [27, 28].

### Context

The study was conducted at University Medical Center Utrecht (UMC Utrecht), The Netherlands. The final year of the Utrecht curriculum is called transitional year and contains elective options and extended clerkships in which students work with increased clinical responsibilities as compared to earlier clerkships [16, 19]. Medical students can enter the transitional year at different times throughout the year, because of delays for any reason during previous study years. The final-year medical student as a semi-physician can be compared to a foundation doctor in the UK system or a sub-intern or intern in the US system [1]. In this final phase, medical students must consider career preferences, to enable choice in the mostly elective final-year program. We deliberately chose participants for our study from this curricular context as they explicitly have the opportunity to work with increased responsibilities in clinical practice during medical school plus one following year and consider their career choice [2, 20].

### Participants

We interviewed the same participants three times over a 2-year period. We chose to plan the first interview at the start of the transitional year, when medical students have the possibility—and are encouraged—to arrange the electives in this year according to their career preference. The second interview was at the end of the transitional year, to explore the impact of this year. The third interview was planned 1 year after graduation, to compare differences in career preference between the medical student perspective and the perspective of the recently graduated physician and explore factors that affect career choice. Dutch students at UMC Utrecht can enter the transitional year at several moments during the academic year. We invited students from one cohort initially during teaching sessions in May and October 2014. All students were also sent an information e-mail and a possibility to sign up. After 1 week, a reminder was sent to all non-responders. We used convenience sampling with the following criteria: being a medical student at Utrecht University at the start of the transitional year of the curriculum during the first round of data collection for this study. No new students were invited in the second and third rounds.

### Instrument

The interview questions were based on what is known from the literature [29, 30]. The first interview guide was piloted with six transitional-year students and was refined prior to implementation in agreement with all researchers. The second and third interview guides were adapted from the first guide in alignment with all researchers. Table 1 presents all three interview guides.

**Table 1:** Interview questions

Start of the final year of medical school (interview 1)	
1) Why did you choose to study medicine? 2) What are your career preferences? Can you explain for each of these why and since when? 3) How familiar are you with these preferences? And what have you done to become familiar with them? 4) What is the opinion of your family, friends or others about your career preference? And what does that opinion mean to you? 5) Which were your electives during the transitional year and why did you choose these? 6) Tell me your strategy to get into the residency of your choice 7) What would be your definite choice just for the upcoming five minutes?	
End of the final year of medical school (interview 2)	
1) What are your career preferences? Can you explain for each of these why and since when? 2) How familiar are you with these preferences? And what have you done to become familiar with them? 3) Can you reflect on your transitional year? 4) What is the opinion of your family, friends or others about your career preference? And what does that opinion mean to you? 5) Tell me your strategy to get into the residency of your choice 6) Did any changes occur in your life of influence on your career preference over the last year? 7) What would be your definite choice just for the upcoming five minutes?	
1 year after graduation (interview 3)	
1) What are your career preferences? Can you explain for each of these why and since when? 2) How familiar are you with these preferences? And what have you done to become familiar with them? 3) Can you reflect on the last year after graduation? 4) Are you working? And does reality matches your expectations? 5) What is the opinion of your family, friends or others about your career preference? And what does that opinion mean to you? 6) Tell me your strategy to get into the residency of your choice 7) Did any changes occur in your life of influence on your career preference over the last year? 8) What would be your definite choice just for the upcoming five minutes? 9) What will be your preference/career in 1.5 years from now?	

Elaboration was encouraged, using follow-up questions about experiences influencing career preferences. To protect the participants’ privacy, all were asked to choose a pseudonym and to retain this for each interview that was used during further analysis. Original names were only known to the interviewers and were not used during analyses.

### Data Collection

We conducted semi-structured, in-depth interviews in a quiet studio at UMC Utrecht to stimulate respondents to mention all aspects that arose without constrictions of a pre-determined questionnaire. The interviews were performed face to face by one of two researchers (SQ and SB) at UMC Utrecht, except for some third interviews performed at the participant’s home or through Skype®. The first three interviews at the start of the final year of medical school were conducted jointly by both researchers, to ensure consistency of interviewing. All interviews were audio recorded. During the interviews, field notes were made for the purpose of reference. Member checking was performed by sharing written summaries of each interview with the participant, with the request to check for accuracy and completeness. During follow-up interviews, participants were not provided with their answers in previous interviews, in order to assure that participants would not justify or adapt their answers based on previously given information.

### Data Analysis

Following a qualitative research methodology, data analysis was performed according to recently suggested standards for reporting qualitative research [26, 28, 31–33]. The interviews were transcribed verbatim using a transcription company with participants’ personal data being de-identified. Two researchers (SQ and SB) first familiarized themselves with the transcripts by multiple readings of the transcripts and checking audio recordings. Next, transcripts were first coded line by line, meaning we identified relevant text passages in the interview transcripts and assigned themes to these data units. Then, these themes were merged in a codebook. The selection of the unit of analysis involved coding for all single factors of influence mentioned by the participants and also coding their career preferences. Coding for these units was performed by reading the transcripts multiple times. For these codes, we (SQ, SB, MdR) created labels that described the essence of the themes. With this process, we identified elements that were similar. This led to an understanding of the variety of factors and the frequency with which they were mentioned. This set of themes could be used for description and interpretation of the phenomenon [28, 31].

For analytical rigour, multiple interviews were coded by three researchers (SQ, SB and MdR). A codebook was developed based on the first interview round and was then discussed with the research team before all data were coded. The codebook was adapted throughout the coding of interview rounds 2 and 3 as needed. We assumed saturation within this sample, when no new insights and no new codes appeared and when the relationships between categories were well established [31, 33]. Researchers (SQ with SB and SQ with MdR) had several meetings to compare their findings of coding and themes, and differences were resolved through discussion. The final codebook and themes were discussed with all research members. All researchers met over several meetings to discuss the coding and analysis. This allowed for alignment of the researchers’ individual interpretations and enhanced reflection, to increase credibility in the content and interpretations of the data [34].

Data analysis started after all the interview had taken place and was further refined in an iterative process with constant comparison and revision of earlier interpretations and developing new themes if needed [31, 35]. Themes emerge naturally from the data rather than being imposed.

### Reflexivity

Reflexivity in qualitative research is a researcher’s ongoing reflection on possible personal biases and being accountable in reporting results [35]. SQ who had no medical background but had knowledge about career options, curricula and the theoretical framework was assigned the role of a researcher with an outsider approach. SB acted as a researcher with an insider approach based on her medical background, her knowledge of the Utrecht medical education program and her recent personal experience herself with medical career choice. This insider/outsider status of the researchers allowed us to understand and interpret the data and to independently perform coding, discuss and resolve any discrepancies. MdR played the role of an outsider based on her knowledge about theoretical frameworks and conducting academic research in general. LW, a family physician by training, fulfilled an insider approach as well as OtC, being a professor of medical education. To maintain reflexivity, the final phases of analyses involved discussions with the larger study team consisting of a mix of insider/outsider approaches and having theoretical knowledge to provide additional perspectives for interpretation. Data analysis continued by importing all data by one researcher (SQ) in a qualitative software application Dedoose® [36].

The study was approved by the Netherlands Association for Medical Education Ethical Review Board. The participants were informed that participation was voluntary, that confidentiality was secured and that non-participation would not be held against them. They could withdraw from the study at any time without giving a reason. Written informed consent was obtained from all the participants.

## Results

The teaching sessions in the first weeks of study year 6 were attended by a total of 67 medical students. A total of 26 students signed up for participation, 24 of whom were able to schedule a first interview and participated. The second interview series involved 22 of these participants and the third interview series 20. We did not know the reasons for withdrawal by those students who failed to participate in the second and third interview processes. One student was able to attend the third interview but skipped the second interview due to medical school–related scheduling conflicts. All 24 first and 22 second interviews were all performed at UMC Utrecht. Of the 20 third interviews, 5 were performed at the participant’s home and 4 through Skype® or telephone, and 11 were performed at UMC Utrecht. One of the first interview series failed to be recorded due to technical problems, yielding 23 interview transcripts. One of the second interview series failed to be recorded due to technical problems, yielding 21 interview transcripts. Of both interviews, field notes and a member check were available.

Member checking of written interview summaries [37] did not lead to essential changes; five participants made minor textual changes in the first round. For the second interview round 2 participants made minor changes and, for the third round, 3 participants did. Participants at the first interview ranged in age from 23 to 26 and included 20 women and 4 men. The second and third interviews included 19 women and 3 men, and 16 women and 4 men, respectively. Some had partners, but there were no married or divorced students; none had children.

The first interview was conducted at the start of the final study year, and all 24 participants were still attending medical school. The second interview was in the weeks before graduation. There were 22 participants, and they were all still medical students. Nine were looking for a job as a PNIT, and four already had the prospect of starting as a PNIT, a total of 13. Three could start residency, three with PhD training and, for three, it was unknown. At the third interview, there were 20 participants and all of them had graduated. Fifteen had a PNIT position, and two had the perspective of starting residency training within 2 months. One had a residency position, three had started a PhD trajectory and one was unemployed. See Table 2. Participants with a PhD position do not work in a clinical setting and could therefore not mention the impact of responsibility for patient care or professional actions on their career choice. We excluded these three from further analyses.

**Table 2:** Background and number of participants at the time of the interview

Interview moment	Beginning of transitional year (interview 1)	End of transitional year (interview 2)	1 year after graduation (interview 3)
In medical school	24	0	0
In a PNIT position*	0	13	15
In residency training	0	3	1
In PhD training	0	3	3
Unemployed/unknown	0	3	1
Total	24	22	20

*Physician-not-in-training position doing clinical work

Nineteen participants completed all three interviews, twenty-two participants participated in interviews 1 and 2 only and one participant participated in interviews 1 and 3 only.

**Fig. 1:**
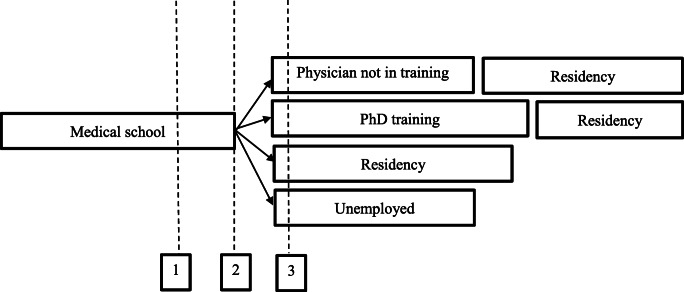
Flowchart of common career paths and times of the interviews (dotted lines)

In Fig. 1, an overview of the different career paths of the study population as also the interview moments are illustrated by the spotted lines. This overview does not cover all possible pathways, as graduates can choose different paths and even can change. We indicated the most common paths, in a schematic fashion

### Career Preferences and Choice

During all three interview rounds, the participants were asked for their career preferences or choice. All participants mentioned their top list of career preferences, ranging from two to five. Two thirds of all participants (17) changed the rank order of their preferences during the interview rounds. Six participants mentioned career preferences they did not had before in interview 3, when they had some experiences as a PNIT. Experiencing responsibilities in patient care was a dominant factor that contributed to the sequence of the top list of career preferences. In the examples below, we explain this further.

### Responsibility as a Doctor

In the interviews, several participants mentioned the dominance of (clinical) responsibility impacting career preference. They explained needing this experience to feel more “certain of their own actions”, to get “more knowledge and experience as a real doctor” and “to experience working in the context of their specialty of preference” to confirm their career preference, as illustrated in three sequential interviews with a female participant (no. 16).

Interview 1: “I did an extra clerkship gynaecology in Africa. In this clerkship, I was allowed to see and participate more, and maybe that is where my career preference was initiated. I thought: this is something I like.

Why? Because you are allowed to see and perform a lot and that is how you develop more knowledge. It makes you more secure in a specific specialty. And when you know it better, you start liking it more”.

Interview 2: “I got more responsibilities this final year clerkship gynaecology and they took me more seriously. I could perform so much, with the responsible doctor just next to me. I learned so much in this final study year, I think I can perform as a doctor”.

Interview 3: “The real work is really something different from what you have seen during your clerkships. In a clerkship, I already felt I had a lot of responsibility and I thought oh yes, I am really in control. But at the end of the day, there is always someone else who makes the decisions. And now, of course, there is still the gynaecologist, and you always consult him. But, I am the doctor now and I have to make acute decisions myself. My career preference is still gynaecology. This year [as a PNIT] I did so much, this confirmed my interest again. In two months, I will start an ObGyn residency and I am really happy about it”.

In the second interview series, participants explained that their insecurity had decreased, due to their responsibility such as performing the full tasks in patient consultations as a semi-physician. These experiences as ‘almost a doctor’ supported them in gaining insight into what it means to perform as a doctor and what it means to be working as a doctor in specific specialty. Several of the participants also mentioned that they looked forward to working as a PNIT and to experience real responsibility for patients and activities and to be more certain of their career choice.

On the third interview, they reflected on being a PNIT and how this resulted in having the full responsibilities of a doctor. They had to make medical decisions, they had to collaborate with and instruct other nursing professionals and they were in charge of threating and caring for patients. The subsequent interviews with a female participant (no. 8) illustrate this.

Interview 1: “If someone calls me a doctor, then I think no I am not a doctor at all. I can’t imagine that I will be a doctor in one year from now”.

Interview 2: “During my clerkship psychiatry, I just got so enthusiastic about everything I did. I like it when patients share their story and that I can help them. Later they came back to say that my suggestions had helped. Maybe that is an idealized view of psychiatry, and that is why I need to experience how psychiatry will be if I work there as a PNIT”.

Interview 3: “The difference between being a semi-physician and PNIT is huge. I felt much more responsible. The patients and colleagues expected so much more from me. Also, the shifts were stressful, sleeping with the beeper that can buzz any moment. I wanted to be a psychiatrist, but after my PNIT period I experienced what this specialty really is about and I miss the somatic part patient care. I returned to my preference for general practice”.

We analysed to what extent additional pre-residency clinical experience and increased responsibilities had affected career choice. In the interviews, the participants explained how responsibilities in patient care, such as autonomously conducting patient consultations and assisting in medical practice, prescribing medicine, collaborating with medical staff and negotiating with families, provided a realistic view of the specialty. For some participants, it was a confirmation of their earlier career preference; for others, the PNIT experience turned out to be disappointing and led them to reconsider their career preference. Of the 15 participants with a PNIT experience, 7 changed their first preference after their experiences as a PNIT. One reason to shift preference was that reality did not match the expected image of the specialty. The opposite also occurred; by gaining more experience within an (unknown) specialty, it could turn out to be more interesting than expected. Interviews with a female participant (no. 12) illustrate this.

Interview 1: “I had a clerkship in family medicine in year 5 and this was a good experience, but I would like to have confirmation that this is really my career choice. That is why a want to go for a family medicine clerkship”.

Interview 2: “My family medicine clerkship was in a small practice with just one general practitioner. I was allowed to perform a lot by myself and it gave me a good insight into a regular day in practice. It confirmed that I really like this specialty”. During her clerkship internal medicine, she experienced more responsibility: “Before I started I was excited and anxious, because I felt I needed to know a lot, you have to bring a lot of knowledge. And, for me that was exciting… but it went really well. That was really positive and maybe it is a cliché, but I thought: I noticed during my clerkship that I am ready for the next step as a PNIT. I hope to become more of a real physician. It is like getting your driving license. You are allowed to drive, but you still have to learn to drive”.

Interview 3: “When I graduated I thought of specializing in family medicine, but I wanted to gain more experience. I started working as a PNIT in geriatrics. I got caught by this specialty. I cannot pinpoint any single moment or occasion how this happened, but the whole experience of working and having the responsibility [in geriatrics] turned out very successful and enjoyable”.

## Discussion

We conducted a longitudinal, interview-based study to explore how students from a vertically integrated curriculum make career choices and whether the phenomenon of pre-residency clinical experiences and increased responsibilities influence this choice.

Our data reveal the importance of responsibility, both when being a medical student and as a PNIT. This responsibility is not confined to any particular specialty and encompasses a realistic experience of becoming and being a physician. This experience encourages the medical student and graduate to reflect on their actions and to translate these reflections into their preferences. With the presence of this phenomenon of ‘responsibility’, a medical student’s or a PNIT’s perception of a specialty is confirmed or changes. The student or PNIT is forced to reflect, (re)consider and possibly adjust their personal career preference. The underlying process of career choice is stimulated by these iterative reflections. This supports the validity of Bland’s model of career choice [29]. This model postulates that the process of choice is essentially a balance between expected future career needs and the perception of the characteristics of a specialty. The stronger the perceived similarity, the stronger the desire for this specialty as a career choice [29]. To achieve a balance between the needs and perceived specialty characteristics, students must actively reflect to establish a career preference(s). We argue that students, and particularly recent graduates, use experience of responsibility to calibrate their perceptions of different specialties’ characteristics with current and future personal needs.

The extent to which responsibility is present in the several phases, in which we interviewed the participants, differs. During medical school, students, even with quite some responsibility, are still limited in their performance as a doctor. Early clerkships enable medical students to experience their chosen profession, helping them to reflect on whether they have chosen the right study [38]. The Dutch medical curricula have evolved, roughly since the turn of the millennium, in a vertically integrated direction [2], not only by planning early clinical experiences [38] but also by creating affordances in the clinical workplace that stress increased responsibility in the higher years [16, 18, 20], such as semi-physician rotations and, recently, the introduction, in some programs, of entrustable professional activities [39]. Our results also show that a clerkship that enables the medical student to work with more responsibility under supervision stimulates him or her to find a balance between the future career needs and the best match with a career preference. We found that pre-residency responsibilities enable medical students to reflect on their career preference in a timely manner. The effect of carrying responsibility is that the graduates can make a well-considered career choice. Not only pre-residency responsibility but also continuing and increased responsibility add to a thoughtful career consideration process. We found that particularly working as a PNIT had a strong effect on career preference considerations. Seven of the 15 graduates who had work experience as a PNIT changed their career choice because of these experiences. Therefore, we should stimulate that final-year students and graduates experience more into the field of the unpopular specialties and get more excited about these specialties. This could lead to a more balanced allocating of the available residency positions. This indicates the relevance of the PNIT period and the effect it can have on the final career choice, supporting the similar findings of Woolf and colleagues [6]. These were limited to the UK, but with our results, we support these statements and think they can be used for countries with different educational systems.

The final career choice is partly based on the experience of independently working as a doctor with responsibility for self-determined actions and patient care. This implies that a medical educational system starting with pre-residency clinical experience and subsequent increase of responsibility during undergraduate training enhances a thoughtful career preference development. Next, graduates significantly benefit from the advantages of working as a PNIT. With this experience as a PNIT, career preference becomes clearer. The difference between the medical student and the PNIT is the license to practice and therefore the level of responsibility for patient care.

## Conclusion

In our study, the responsibility to work as a physician, i.e. being more self-directed than as a medical student, *before* postgraduate training, appears to significantly affect career choice. Practicing with responsibility as a PNIT provides one’s realistic view on work impact and one’s reflection of their future self-image and therefore enables to match with their career preference and to make their career choice.

## Strengths and Limitations of This Study

This study was performed in the context of one Dutch medical school. Other schools and particularly other countries may show different results. The proportion of female students in Dutch medical schools (65%) [10, 40] is lower than that in our study population (83%) which could have caused a bias in the data. Previous research shows that men and women differ in the motivational factors that are associated with specialty preference, regarding part-time work, status and salary [41, 42]. Nevertheless, the stories of the male students in our study showed similar experience with clinical responsibility and the importance for their career choice. Future research, particularly quantitative with a large sample, is needed to substantiate our results to counteract to the physician workforce of today.

## Abbreviation

PNIT: Physician-not-in-training

## References

[1] Wijnen-Meijer M, Burdick W, Alofs L, Burgers C, ten Cate O (2013). Stages and transitions in medical education around the world: clarifying structures and terminology. Med Teach..

[2] Ten Cate O (2007). Medical education in the Netherlands. Med Teach..

[3] Hoff R, Imhof S, Frenkel F, ten Cate OTJ (2018). Flexibility in postgraduate medical training in the Netherlands. Acad Med..

[4] van der Vleuten C, Scherpbier A (2009). AM Last Page: medical education in the Netherlands. Acad Med..

[5] van der Velde F, Wierenga M. Loopbanen en loopbaanwensen van basisartsen. Herhaling van het onderzoek onder basisartsen 2009 en 2012. Kiwa. 2016. [In Dutch]

[6] Woolf K, Elton C, Newport M (2015). The specialty choices of graduates from Brighton and Sussex Medical School: a longitudinal cohort study. BMC Med Educ..

[7] Andlauer O, Guicherd W, Haffen E, Sechter D, Bonin B, Seed K (2012). Factors influencing French medical students towards a career in psychiatry. Psychiatr Danub..

[8] Baller FA, Ludwig KV, Kinas-Gnadt Olivares CL, Graef-Calliess I-T (2013). Exploring the ideas and expectations of German medical students towards career choices and the speciality of psychiatry. Int Rev Psychiatry.

[9] Hou J, Xu M, Kolars JC, Dong Z, Wang W, Huang A (2016). Career preferences of graduating medical students in China: a nationwide cross-sectional study. BMC Med Educ.

[10] Pfarrwaller E, Sommer J, Chung C, Maisonneuve H, Nendaz M, Junod Perron N (2015). Impact of interventions to increase the proportion of medical students choosing a primary care career: a systematic review. J Gen Intern Med..

[11] Capaciteitsorgaan. The 2016 recommendations for medical specialist training. Utrecht; 2016. [In Dutch]

[12] Capaciteitsorgaan. Personal communication with director V. Slenter. 2017. [In Dutch]

[13] Wijnen-Meijer M. Readiness for clinical practice—studies about transitions in medical education, the influence of vertically integrated curricula and the assessment of readiness for practice [doctoral dissertation]. Utrecht, The Netherlands: Utrecht University; 2012.

[14] Dornan T, Bundy C (2004). What can experience add to early medical education?. Consensus survey. BMJ..

[15] ten Cate OTJ, Borleffs JCC, Van Dijk M, Westerveld T. Training medical students for the 21st century: rationale and development of the Utrecht curriculum “CRU+.” Med Teach. 2018. 10.1080/0142159X.2018.1435855 29468920

[16] Jonker G, Hoff RG, Max S, Kalkman CJ, ten Cate O (2017). Connecting undergraduate and postgraduate medical education through an elective EPA-based transitional year in acute care: an early project report. GMS J Med Educ..

[17] Wijnen-Meijer M, Ten Cate OTJ, Van Der Schaaf M, Harendza S (2013). Graduates from vertically integrated curricula. Clin. Teach..

[18] Wijnen-Meijer M, Ten Cate OTJ, Van Der Schaaf M, Burgers C, Borleffs J, Harendza S (2015). Vertically integrated medical education and the readiness for practice of graduates. BMC Med. Educ..

[19] van den Broek WES, Wijnen-Meijer M, Ten Cate O, van Dijk M (2017). Medical students’ preparation for the transition to postgraduate training through final year elective rotations. GMS J Med Educ.

[20] Wijnen-Meijer M, Ten Cate OTJ, Van Der Schaaf M, Borleffs JCC (2010). Vertical integration in medical school: effect on the transition to postgraduate training. Med Educ..

[21] Wijnen-Meijer M, Ten Cate OTJ, Rademakers JJDJM, Van Der Schaaf M, Borleffs JCC (2009). The influence of a vertically integrated curriculum on the transition to postgraduate training. Med Teach..

[22] Goldin SB, Schnaus MJ, Horn G, Mateka J, DiGennaro J, Wahi M (2012). Surgical interest and surgical match for third-year students: results of a prospective multivariate longitudinal cohort study. J Am Coll Surg..

[23] Compton MT, Frank E, Elon L, Carrera J (2008). Changes in U.S. medical students’ specialty interests over the course of medical school. J Gen Intern Med..

[24] Goldacre MJ, Laxton L, Lambert TW (2010). Medical graduates’ early career choices of specialty and their eventual specialty destinations: UK prospective cohort studies. BMJ..

[25] Maudsley G, Williams L, Taylor D, Maudsley G, Williams LYN, Taylor D (2010). Medical students’ and prospective medical students’ uncertainties about career intentions: cross-sectional and longitudinal studies. Med Teach.

[26] Balmer DF, Richards BF (2017). Longitudinal qualitative research in medical education. Perspect Med Educ..

[27] Husserl E. Ideas: general introduction to pure phenomenology. New York: Collier Books; 1962.

[28] Swanwick T. Understanding medical education: evidence, theory and practice. J Public Health. 2014.

[29] Bland C, Meurer L, Maldona G (1995). Determinants of primary care specialty choice; a non-statistical meta-analysis of the literature. Acad Med.

[30] Querido SJ, Vergouw D, Wigersma L, Batenburg RS, De Rond MEJ, Ten Cate OTJ (2015). Dynamics of career choice among students in undergraduate medical courses. A BEME systematic review: BEME Guide No. 33. Med Teach..

[31] Creswell J. Research design: qualitative, quantitative, and mixed methods approaches. Michigan: Sage Publications Inc.; 2014.

[32] O’Brien BC, Harris IB, Beckman TJ, Reed DA, Cook DA (2014). Standards for reporting qualitative research: a synthesis of recommendations. Acad Med..

[33] Miles MB, Huberman M a, Saldana J. Qualitative data analysis. A methods sourcebook. 3rd edn. Qualitative data analysis: a methods sourcebook. Arizona: Sage Publications Inc.; 2014.

[34] Cook DA, Kuper A, Hatala R, Ginsburg S (2016). When assessment data are words. Acad Med.

[35] Malterud K (2001). Qualitative research: standards, challenges, and guidelines. Lancet..

[36] SocioCultural Research Consultants L. Dedoose, version, 7.0.23. web application for managing, analyzing, and presenting qualitative and mixed method research data [Internet]. Los Angeles; 2016. Available from: www.dedoose.com

[37] Tavakol M, Sandars J (2014). Quantitative and qualitative methods in medical education research: AMEE Guide No. 90: Part II. Med Teach..

[38] Kamalski DMA, Ter Braak EWMT, Ten Cate OTHJ, Borleffs JCC (2007). Early clerkships. Med Teach..

[39] Ten Cate OTJ, Graafmans L, Posthumus I, Welink L, Van Dijk M (2018). The EPA-based Utrecht undergraduate clinical curriculum: development and implementation. Med. Teach..

[40] Capaciteitsorgaan. The 2013 recommendations for medical specialist training. Utrecht; 2013. [In Dutch]

[41] Diderichsen S, Johansson EE, Verdonk P, Lagro-Janssen T, Hamberg K (2013). Few gender differences in specialty preferences and motivational factors: a cross-sectional Swedish study on last-year medical students. BMC Med Educ..

[42] Heikkilä TJ, Hyppölä H, Vänskä J, Halila H, Kujala S, Virjo I (2016). What predicts doctors’ satisfaction with their chosen medical specialty? A Finnish national study. BMC Med Educ..

